# Targeting DNA repair with aphidicolin sensitizes primary chronic lymphocytic leukemia cells to purine analogs

**DOI:** 10.18632/oncotarget.9525

**Published:** 2016-05-20

**Authors:** Eliza Starczewska, Maxime Beyaert, Lucienne Michaux, Marie-Christiane Vekemans, Pascale Saussoy, Vanesa Bol, Ainhoa Arana Echarri, Caroline Smal, Eric Van Den Neste, Françoise Bontemps

**Affiliations:** ^1^ de Duve Institute, Université Catholique de Louvain, B-1200 Brussels, Belgium; ^2^ Department of Hematology, Cliniques Universitaires Saint-Luc, Université Catholique de Louvain, B-1200 Brussels, Belgium; ^3^ Service de Biologie Clinique, Cliniques Universitaires Saint-Luc, Université Catholique de Louvain, B-1200 Brussels, Belgium; ^4^ Center for Molecular Imaging, Radiotherapy and Oncology, Institut de Recherche Experimentale et Clinique (IREC), Université Catholique de Louvain, B-1200 Brussels, Belgium

**Keywords:** chronic lymphocytic leukemia, DNA damage, fludarabine, aphidicolin, γH2AX

## Abstract

Purine analogs are among the most effective chemotherapeutic drugs for the treatment of chronic lymphocytic leukemia (CLL). However, chemoresistance and toxicity limit their clinical use. Here, we report that the DNA polymerase inhibitor aphidicolin, which displayed negligible cytotoxicity as a single agent in primary CLL cells, markedly synergizes with fludarabine and cladribine *via* enhanced apoptosis. Importantly, synergy was recorded regardless of CLL prognostic markers. At the molecular level, aphidicolin enhanced purine analog-induced phosphorylation of p53 and accumulation of γH2AX, consistent with increase in DNA damage. In addition, aphidicolin delayed γH2AX disappearance that arises after removal of purine analogs, suggesting that aphidicolin causes an increase in DNA damage by impeding DNA damage repair. Similarly, aphidicolin inhibited UV-induced DNA repair known to occur primarily through the nucleotide excision repair (NER) pathway. Finally, we showed that fludarabine induced nuclear import of XPA, an indispensable factor for NER, and that XPA silencing sensitized cell lines to undergo apoptosis in response to fludarabine. Together, our data indicate that aphidicolin potentiates the cytotoxicity of purine analogs by inhibiting a DNA repair pathway that involves DNA polymerases, most likely NER, and provide a rationale for manipulating it to therapeutic advantage.

## INTRODUCTION

Chronic lymphocytic leukemia (CLL) is a clonal lymphoproliferative disorder characterized by the progressive accumulation of mature, typically CD5/CD19-positive B cells, most of which are in the G0/G1 phase of the cell cycle [1, 2]. Despite great advances in the management of CLL with the introduction of targeted drugs, such as ibrutinib and idelalisib [3], chemoimmunotherapy with the purine analog fludarabine in combination with cyclophosphamide and the anti-CD20 monoclonal antibody rituximab (FCR) remains the standard first-line treatment for CLL [4]. However, except for some selected patients with favorable characteristics [5], nearly all patients eventually relapse post-FCR. Therefore, identification of therapies with novel mechanisms of action, particularly agents that could complement existing therapeutic options, remains a priority.

Purine analogs like fludarabine and cladribine are prodrugs that require intracellular conversion into triphosphate derivatives to be active. Triphosphate analogs have several mechanisms of action, which collectively lead to induction of DNA strand breaks, p53 up-regulation and apoptosis [6]. The first and rate-limiting step of purine analog activation is catalyzed by deoxycytidine kinase (dCK), an enzyme that plays a critical role in purine analog efficacy [7]. We previously demonstrated that several genotoxic agents, including UV-irradiation and DNA synthesis inhibitors, such as aphidicolin, enhance dCK activity in leukemic cells by inducing phosphorylation of dCK at Ser-74 [8, 9]. This finding led us to postulate that combination of nucleoside analogs with drugs like aphidicolin might enhance their conversion into nucleoside analog triphosphate and thereby their effectiveness. However, increase of dCK activity in CLL cells through Ser-74 phosphorylation was found to augment the activation of pyrimidine analogs, but not of purine analogs [10]. Yet, we observed in preliminary experiments that aphidicolin potentiated the cytotoxicity of fludarabine and cladribine in primary CLL cells, suggesting a still unidentified mechanism of sensitization to purine analogs.

Aphidicolin is a natural tetracyclic diterpene that inhibits DNA polymerases α, δ, and ε, and therefore DNA replication and certain forms of DNA repair [11]. Especially nucleotide excision repair (NER), which requires DNA polymerases δ and ε for the final step of the repair, is sensitive to aphidicolin [12]. This property of aphidicolin has provided the rationale for combining it with DNA-damaging anticancer drugs whose effectiveness could be limited by activation of NER. In particular, aphidicolin has been shown to enhance the potency of cisplatin in primary ovarian tumors and of chlorambucil in CLL lymphocytes [13–15]. Also, it was found to potentiate the cytotoxicity of the pyrimidine analog cytarabine in blast cells from adult and pediatric patients with acute myeloid leukemia, whereas it did not modify sensitivity of the same cells to fludarabine [16, 17].

In the present study, we aimed to analyze the combination aphidicolin/fludarabine or aphidicolin/cladribine in CLL samples of a larger cohort of patients and determine the mechanism by which aphidicolin could increase purine analog cytotoxicity. Particularly, we assessed whether inhibition of DNA repair could underlie the beneficial effect of this combination in primary CLL cells.

## RESULTS

### Aphidicolin enhances sensitivity of primary CLL cells to fludarabine and cladribine

**Figure 1:**
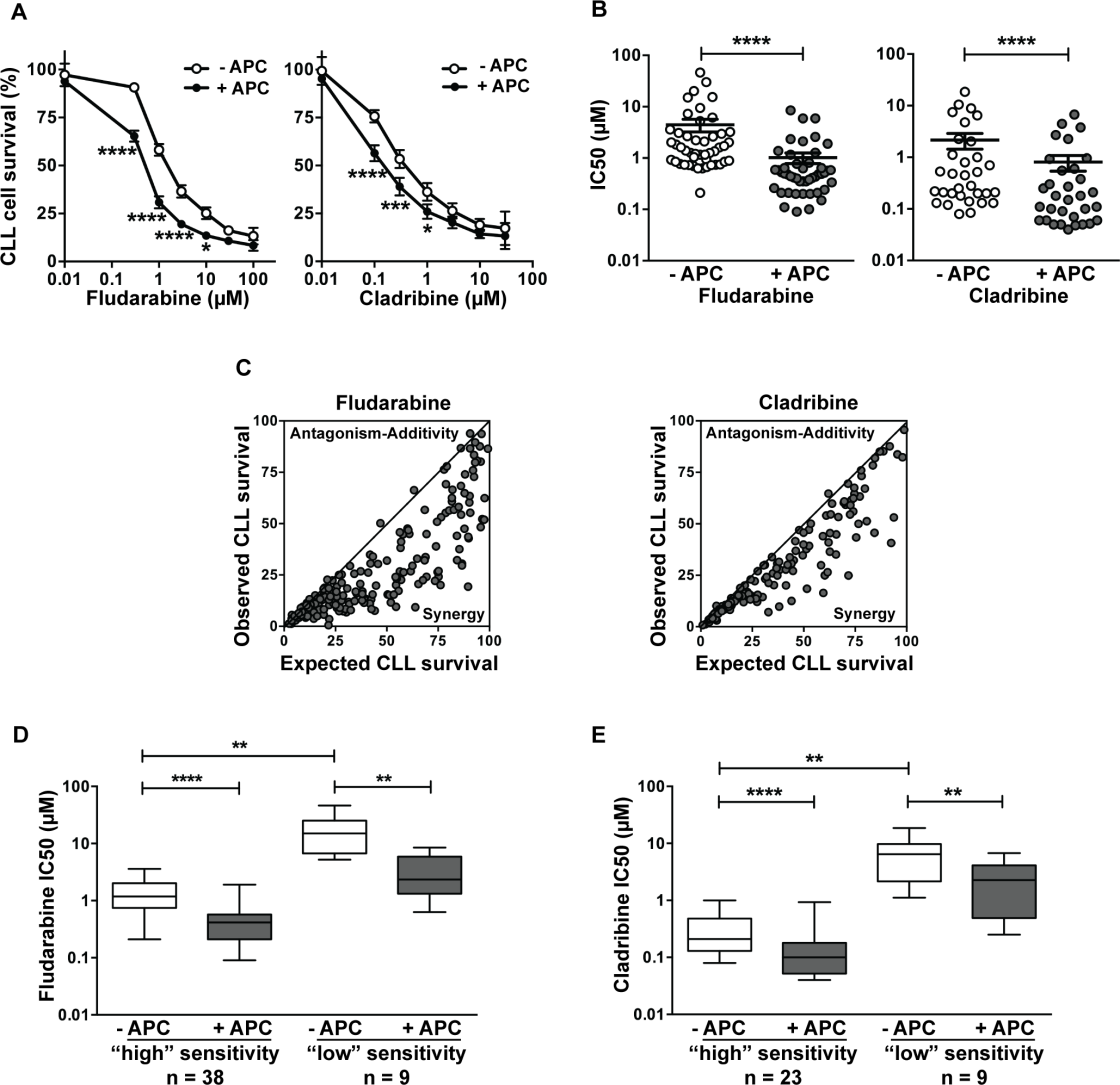
Aphidicolin enhances fludarabine and cladribine cytotoxicity in primary CLL lymphocytes CLL lymphocytes were incubated in the presence or absence of 3 μM aphidicolin (APC) and various concentrations of fludarabine or cladribine for 96 h. Cell viability was measured using the MTT assay. Forty-seven and thirty-two analyses were performed for fludarabine and cladribine, respectively. (**A**) Dose-response curves of fludarabine or cladribine in the absence or presence of APC. Values shown are means ± SEM. Significance relative to the absence of APC was analyzed by two-way Anova followed by Bonferroni post-test. (**B**) IC_50_ of fludarabine and cladribine in the absence or presence of APC calculated from the individual data of Figure 1A. Individual IC_50_ and means ± SEM are shown. Significance relative to the absence of APC was analyzed by Wilcoxon matched-pairs signed-rank test. (**C**) Comparison between observed and expected cell survival for the combination fludarabine/APC and cladribine/APC was performed according to the multiplicative model [18]. The continuous line is *x* = *y*. (**D**–**E**) Effect of aphidicolin according to the sensitivity of CLL cells to purine analogs. CLL samples were divided into two groups (of “high” or “low” sensitivity) on the base of the IC_50_ of fludarabine (D) or cladribine (E) obtained in the absence of APC: < or ≥ 5 μM for fludarabine, and < or ≥ 1 μM for cladribine. Significance relative to the absence of APC was analyzed by Wilcoxon matched-pairs signed-rank test and difference between less and more sensitive samples was assessed by Mann-Whitney test. For all panels: *P < 0.05; **P < 0.01; ***P < 0.001; ****P < 0.0001.

Cytotoxicity of aphidicolin and purine analogs, alone or in combination, was assessed using the MTT assay. Aphidicolin was used at 3 μM, a concentration that was found to increase dCK activity by 2- to 3-fold in CLL cells [9, 10]. At this concentration, aphidicolin alone induced negligible cytotoxicity, the loss of CLL cell viability being 5.6 ± 0.9 % after 4 days (*n* = 47). Overall, we investigated the combination aphidicolin/fludarabine in 47 samples from 29 individual CLL patients and the combination aphidicolin/cladribine in 32 samples from 21 patients. As illustrated in Figure 1A, the DNA polymerase inhibitor significantly increased CLL cell sensitivity to fludarabine and cladribine. On average, aphidicolin decreased the IC_50_ of fludarabine from 4.5 ± 1.2 to 1.0 ± 0.2 μM (*n* = 47) and that of cladribine from 2.2 ± 0.7 to 0.8 ± 0.3 μM (*n* = 32), thus increasing CLL cell sensitivity to fludarabine and cladribine by 4.5- and 2.8-fold, respectively (Figure 1B). Sensitization by aphidicolin (defined as the ratio of the IC_50_ obtained in the absence or presence of aphidicolin) ranged from 1 to 21.9 for fludarabine and from 1 to 8.2 for cladribine, being ≥ 2 in 39/47 analyses (83% of patients) for fludarabine and in 19/32 analyses (59% of patients) for cladribine. In only one of the 29 CLL samples, aphidicolin had no sensitizing effect for either of the analogs. Interaction of aphidicolin with fludarabine or cladribine, analyzed according to the multiplicative model [18], was found to be synergistic in almost all conditions (Figure 1C).

As the sensitivity to fludarabine or cladribine widely varied among patients, we split the cohort into two subsets, one “more sensitive” and the other “less sensitive” to the drug, as determined by the choice of an arbitrary threshold IC_50_ value: 5 μM for fludarabine (Figure 1D) and 1 μM for cladribine (Figure 1E). Comparison showed that the sensitizing effect of aphidicolin was present in the two subgroups.

### Sensitization by aphidicolin is independent of the clinical parameters of CLL patients

The sensitizing effect of aphidicolin was analyzed in relation with the clinical characteristics of the patients. CLL samples were stratified in different groups based on the *IGHV* mutational status, the treatment (untreated *versus* treated) and cytogenetics (17p deletion *versus* no deletion). Cytotoxicity of fludarabine was significantly lower in CLL cells from unmutated or previously treated than in mutated or untreated patients, but not significantly different between 17p deleted and non-deleted cases (Table 1A). Sensitization by aphidicolin was observed in each subtype, being very similar in samples from patients with good or poor prognostic features. Concerning cladribine, CLL cells from unmutated, pretreated or 17p deleted patients were less sensitive to the drug than their counterparts, but the difference was only significant between untreated and pretreated patients (Table 1B). Sensitization by aphidicolin was also noted in each subgroup. No correlation with CD38 expression or 11q deletion could be performed, due to insufficient number of patients with these characteristics.

**Table 1:** Correlation between sensitization by aphidicolin and clinical parameters

(A) Fludarabine			
Characteristics (*n*)	IC_50_ (μM)		Sensitization ratio (−APC/+APC)
	−APC	+APC	
*IGHV*			
Mutated (15)	2.8 ± 0.5	0.7 ± 0.1	4.0^****^
Unmutated (6)	19.1 ± 7.1^b^	3.3 ± 1.3^b^	5.8^*^^ns^
Previous treatments			
No (30)	2.1 ± 0.5	0.5 ± 0.1	4.2^****^
Yes (14)	10.3 ± 3.6^a^	2.2 ± 0.7a	4.7^***^^ns^
17p deletion			
Non-deleted (29)	5.2 ± 1.9	1.1 ± 0.3	4.7^****^
Deleted (6)	5.4 ± 2.3^ns^	1.6 ± 0.9^ns^	3.4^*^^ns^

(B) Cladribine			
Characteristics (*n*)	IC_50_ (μM)		Sensitization ratio (−APC/+APC)
	−APC	+ APC	
*IGHV*			
Mutated (12)	2.2 ± 1.0	0.9 ± 0.4	2.4^***^
Unmutated (5)	3.4 ± 2.0^ns^	2.1 ± 1.2^ns^	1.6^*^^ns^
Previous treatments			
No (21)	1.0 ± 0.4	0.3 ± 0.1	3.3^****^
Yes (10)	4.8 ± 2.0^a^	1.9 ± 0.7^b^	2.5^**^^ns^
17p deletion			
Non-deleted (21)	2.0 ± 1.0	0.7 ± 0.3	2.9^****^
Deleted (5)	6.3 ± 3.3^ns^	0.9 ± 0.4^ns^	7.0^**^^a^

When possible, CLL samples were classified according to known clinical prognostic markers and sensitization by aphidicolin (APC) was compared between the matched subgroups. IC_50_ values obtained for fludarabine (A) and cladribine (B) in the absence or presence of 3 μM APC are means ± SEM. Significance relative to the absence of APC was analyzed by paired *t*-test or Wilcoxon matched-pairs signed rank test:

* *P* < 0.05;

** *P* <0.01;

*** *P* < 0.001;

**** *P* < 0.0001.

Significance relative to *IGHV* status, previous treatment or 17p deletion was analyzed by unpaired *t*-test with Welch's correction:

a *P* <0.05;

b *P* < 0.01; ns: not significant.

### Aphidicolin enhances apoptosis induced by purine analogs

**Figure 2:**
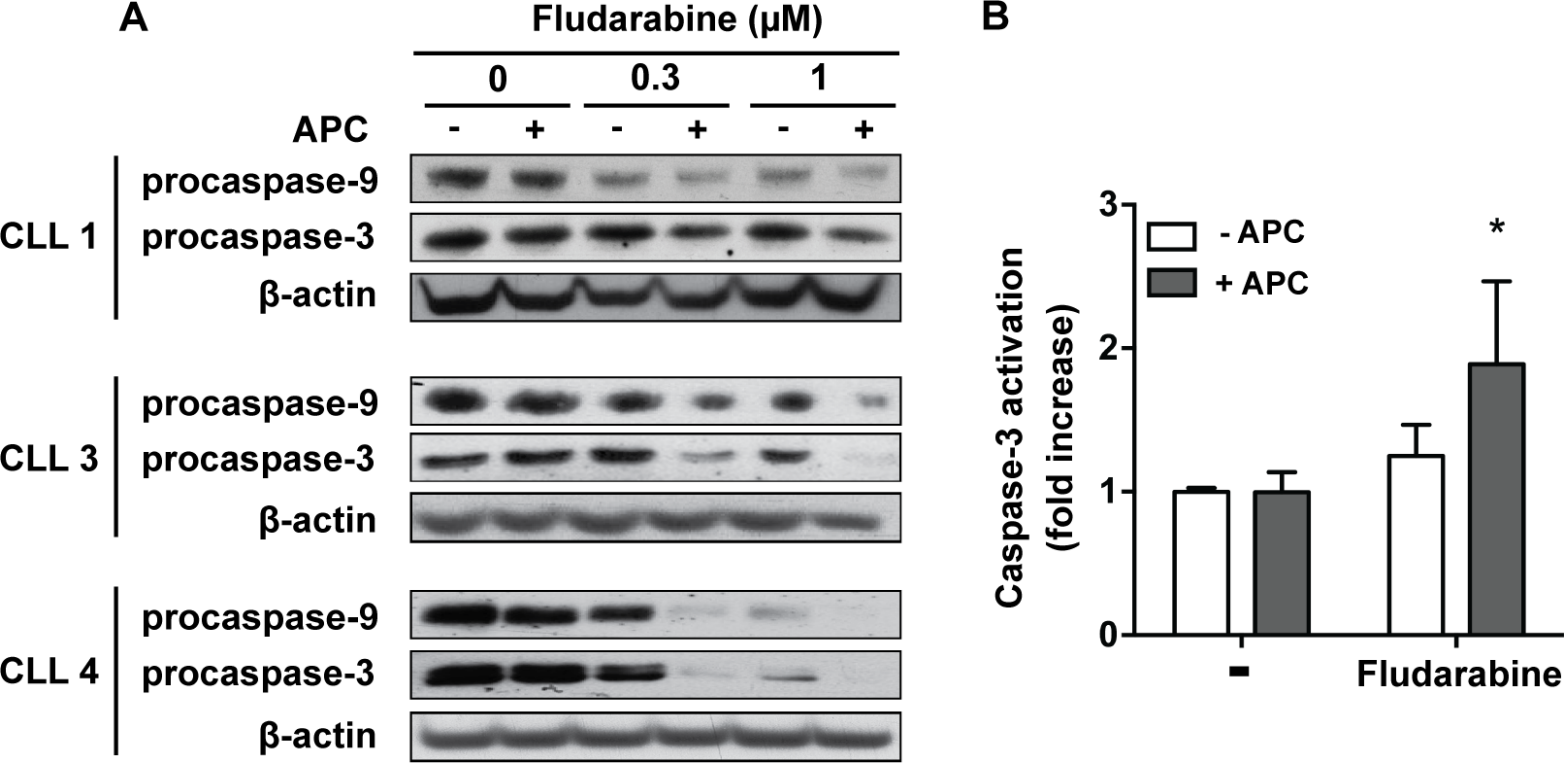
Aphidicolin enhances fludarabine-induced apoptosis (**A**) Procaspase-9 and procaspase-3 were analyzed by Western blot in CLL cells from three patients after a 48 h-incubation in the absence or presence of 3 μM aphidicolin (APC) with or without fludarabine at 0.3 and 1 μM. β-Actin served as a loading control. (**B**) Caspase-3 activity was measured by a fluorometric assay in CLL cells after a 24 h-incubation in the absence or presence of 3 μM APC with or without 1 μM fludarabine (*n* = 4). Values are means ± SEM. Significance relative to the absence of APC was analyzed by two-way Anova followed by Bonferroni post-test: *P < 0.05.

Activation of apoptosis through the intrinsic pathway is considered as the major mechanism of cytotoxicity of purine analogs [6]. Analysis of cell death by flow cytometry using FITC-Annexin V and PI staining (Supplementary Figure 1) confirmed that fludarabine induces apoptosis in CLL cells, most of cells being Annexin-V^+^/PI^−^ after 48 h of treatment, and that aphidicolin could increase fludarabine-induced apoptosis. In accordance with these results, we observed that cleavage of procaspase-9 and procaspase-3, which are crucial events for the initiation of apoptosis by the intrinsic pathway, were enhanced when aphidicolin was combined with fludarabine (Figure 2A). We could not detect the active cleaved form of caspase-3 by Western blot, but a fluorometric assay confirmed that caspase-3 activity was significantly higher when fludarabine was combined with aphidicolin (Figure 2B). Similar results were obtained with cladribine (not shown).

### Aphidicolin increases DNA damage in response to fludarabine and cladribine

**Figure 3:**
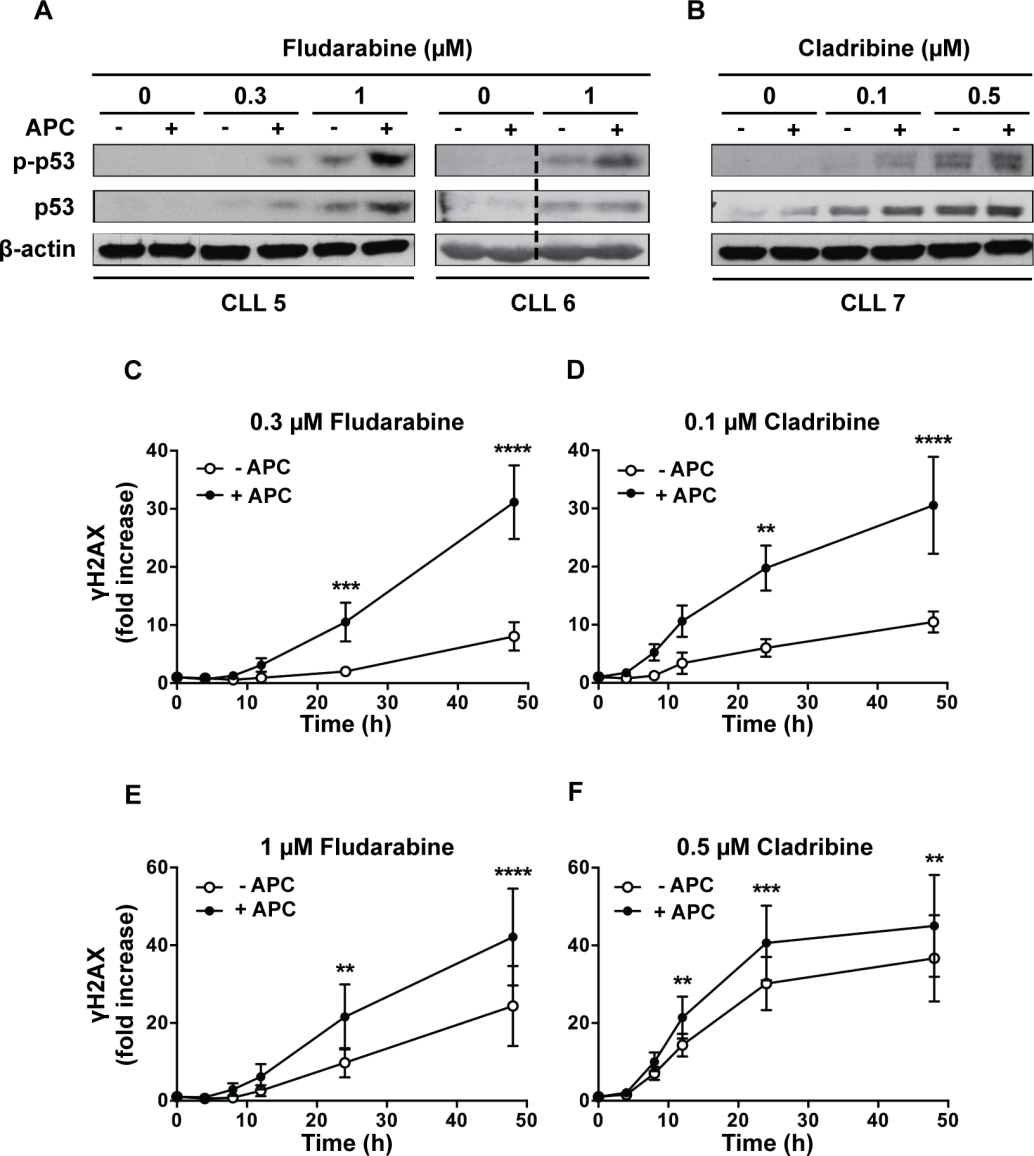
Effect of aphidicolin on p53 phosphorylation and γH2AX accumulation induced by purine analogs (**A**–**B**) CLL cells from 3 different patients were incubated for 24 h with fludarabine (A) or cladribine (B) at the indicated concentrations in the absence or presence of 3 μM aphidicolin (APC). Phosphorylation of p53 at Ser-15 and total p53 were analyzed by Western blot. Data shown for CLL6 were cropped from the same western blot image and combined as indicated by the dotted line. (**C**–**F**) Time-course accumulation of γH2AX in CLL cells incubated with 0.3 or 1 μM fludarabine (C, E) or with 0.1 or 0.5 μM cladribine (D, F) in the absence or presence of 3 μM aphidicolin (APC). Results are means ± SEM of 5 independent experiments. Significance relative to the absence of APC was analyzed by two-way Anova followed by Bonferroni post-test: **P < 0.01; ***P < 0.001; ****P < 0.0001. In all panels, γH2AX is expressed as fold increase over untreatred cells.

To unravel the mechanisms underlying synergy between aphidicolin and purine analogs, we investigated the activation of p53, a key molecular event in chemotherapy using DNA-damaging agents. Whereas aphidicolin alone showed no effect, it increased the phosphorylation of p53 at Ser-15 induced by fludarabine and cladribine (Figure 3A, 3B). Similar results were obtained for p53 protein level, except in the CLL6 sample in which no effect of aphidicolin was noticed.

We also analyzed the phosphorylation of histone H2AX on Ser-139, defined as γH2AX, which occurs shortly after DNA strand breaks and is considered as a surrogate marker of early DNA damage [19, 20]. A dose-dependent increase of γH2AX was clearly detected after incubation with fludarabine or cladribine for 24 h (Supplementary Figure 2). We then examined the influence of aphidicolin on the time-course accumulation of γH2AX at two clinically achievable concentrations of fludarabine (Figure 3C, 3E) or cladribine (Figure 3D, 3F). A latency period (varying between 4 and 12 h depending on the analog concentration) was observed, most probably due to the time required for accumulation of sufficient genotoxic damage. Aphidicolin, which alone did not cause significant γH2AX accumulation (data not shown), reduced this latency and markedly enhanced H2AX phosphorylation induced by fludarabine and cladribine, especially at lower purine analog concentrations (Figure 3C, 3D). These data corroborated that aphidicolin enhances the DNA damage caused by purine analogs.

Using fludarabine at 1 μM and cladribine at 0.1 μM, we verified that a 24 h-incubation in the presence of aphidicolin modified neither their accumulation under triphosphate derivatives, nor their incorporation into nucleic acids (results not shown), confirming our previous results at shorter time points [10]. We thus deduced that increase of purine analog-induced DNA damage by aphidicolin could not be explained by higher activation of these drugs. Finally, we tested the effect of aphidicolin on the cytotoxicity of chemotherapeutic drugs that do not require activation by dCK for their efficacy (Supplementary Table 1). We found that aphidicolin markedly potentiated the cytotoxicity of mafosfamide and doxorubicin in CLL cells, similarly to what was reported previously for chlorambucil [14]. In contrast, aphidicolin did not significantly increase the cytotoxicity of nutlin-3a that acts independently of DNA damage [21].

### Aphidicolin impedes the repair of purine analog-induced DNA damage

**Figure 4:**
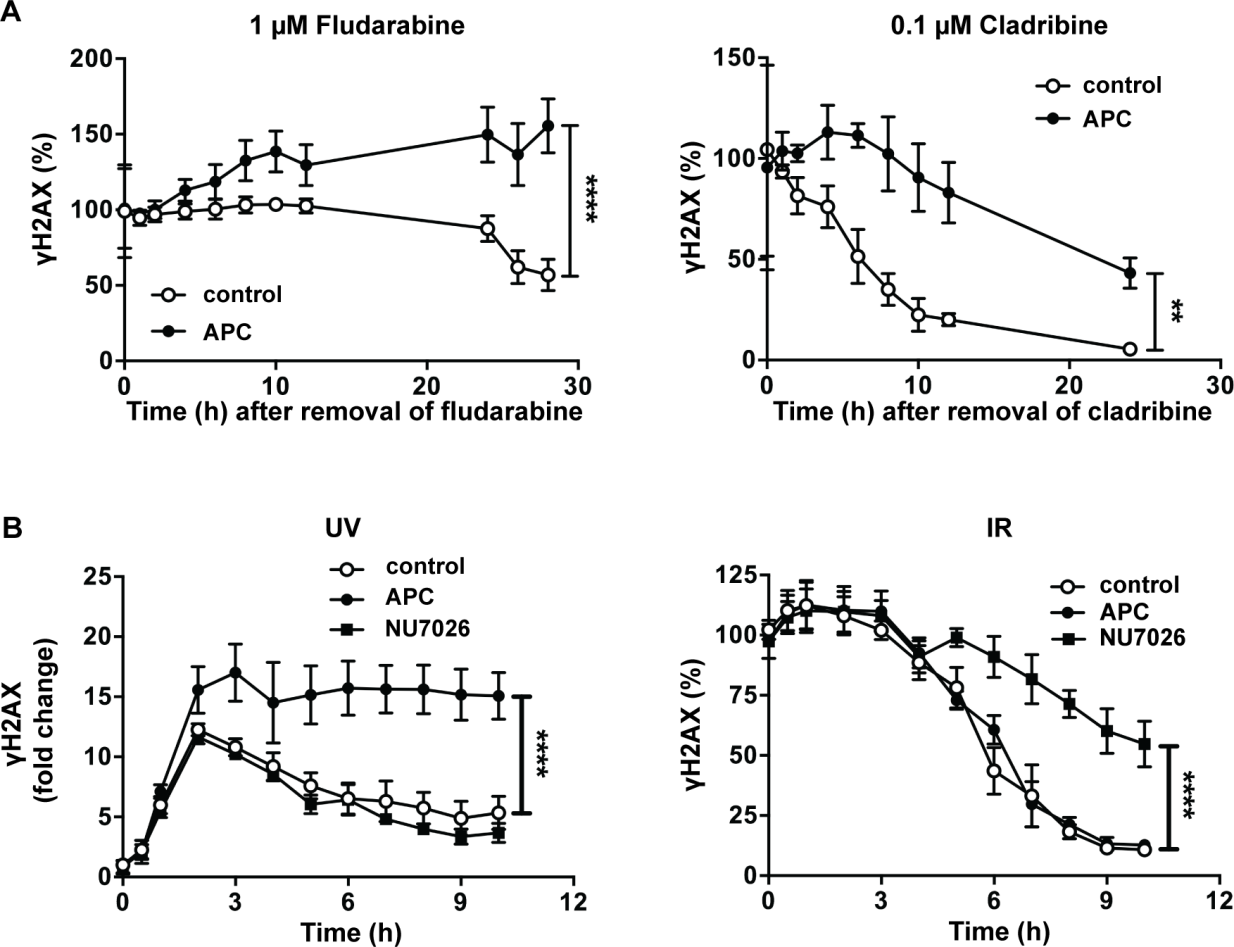
Effect of aphidicolin on DNA repair after various DNA damaging treatments (**A**) CLL cells were preincubated for 24 h with 1 μM fludarabine (left panel) or 0.1 μM cladribine (right panel). Cells were then washed, resuspended in fresh medium and incubated in the absence or presence of 3 μM aphidicolin (APC). γH2AX was analyzed by flow cytometry at the indicated times and expressed as % of the value at time 0. (**B**) CLL cells were subjected to UV-C light (30 J/m^2^) (left panel) or IR at a dose of 5 Gy (right panel) and immediately incubated in the absence or presence of 3 μM APC or 10 μM NU7026. γH2AX was analyzed by flow cytometry at the indicated times and expressed as fold change (UV) or as % of the value at time 0 (IR). In all panels, results are means ± SEM of 3–5 independent experiments. Significance relative to the absence of APC (shown at the last incubation time) was analyzed by two-way Anova followed by Bonferroni post-test: **P < 0.01; ****P < 0.0001.

We investigated whether aphidicolin could increase DNA damage induced by purine analogs through inhibition of DNA repair. CLL cells were incubated for 24 h in the presence of 1 μM fludarabine or 0.1 μM cladribine in order to induce γH2AX accumulation. Then, cells were washed to remove purine analogs, resuspended in fresh culture medium and incubated for several hours in the presence or absence of aphidicolin. In cells pretreated with fludarabine (Figure 4A, left panel) and incubated in the absence of aphidicolin, γH2AX level remained stable for about 12 h before declining gradually, consistent with a progressive repair of the DNA damage induced by fludarabine. In contrast, the presence of aphidicolin provoked additional accumulation of γH2AX, which was maintained over time at significantly higher level than in its absence. In cells pretreated with cladribine (Figure 4A, right panel), γH2AX decrease was also markedly slowed down by aphidicolin. These results indicate that aphidicolin inhibits DNA repair after exposure to purine analogs.

### Aphidicolin inhibits DNA repair induced by UV-C, but not by ionizing radiation

Next step was to identify the DNA repair pathway inhibited by aphidicolin in response to purine analogs. The DNA polymerases δ and ε, which are inhibited by aphidicolin, are implicated in the DNA resynthesis step of various forms of DNA repair, comprising NER, mismatch repair (MMR) and long-patch base excision repair (BER), but not short-patch BER that only employs polymerase β [22]. Long-patch BER and MMR prominently handle repair during S and G2 phase of the cell cycle, whereas NER is active in both dividing and non-dividing cells [23]. Since CLL cells are non-dividing *in vitro,* we reasoned that NER could be the repair pathway targeted by aphidicolin. To substantiate this hypothesis, we analyzed the effect of aphidicolin on DNA damage repair after UV-C irradiation, a treatment known to activate NER in CLL cells [24, 25]. As illustrated in Figure 4B (left panel), UV-C irradiation triggered γH2AX accumulation that reached a maximal level after 2 h and was higher in the presence of aphidicolin. Thereafter, γH2AX progressively decreased in cells incubated in the absence of aphidicolin, whereas it was maintained at its maximal levels in its presence, indicating that repair of the UV-C-induced DNA damage was inhibited. For comparison, we performed the same type of experiments after ionizing radiation (IR) (Figure 4B, right panel). IR mainly activates the non-homologous end joining (NHEJ) pathway, which does not involve DNA polymerases δ or ε, but polymerases μ and λ [26]. H2AX phosphorylation induced by IR (5 Gy) was almost maximal at the first sampling time and started to decrease after 3 h consistent with progressive DNA repair. As expected, aphidicolin did not influence repair of DNA damage induced by IR in contrast with NU7026 that inhibits DNA-dependent protein kinase, a key actor of NHEJ [27]. We verified that NU7026 did not inhibit DNA repair after UV-C-irradiation (Figure 4B, left panel).

### Fludarabine induces nuclear import of XPA in CLL cells

**Figure 5:**
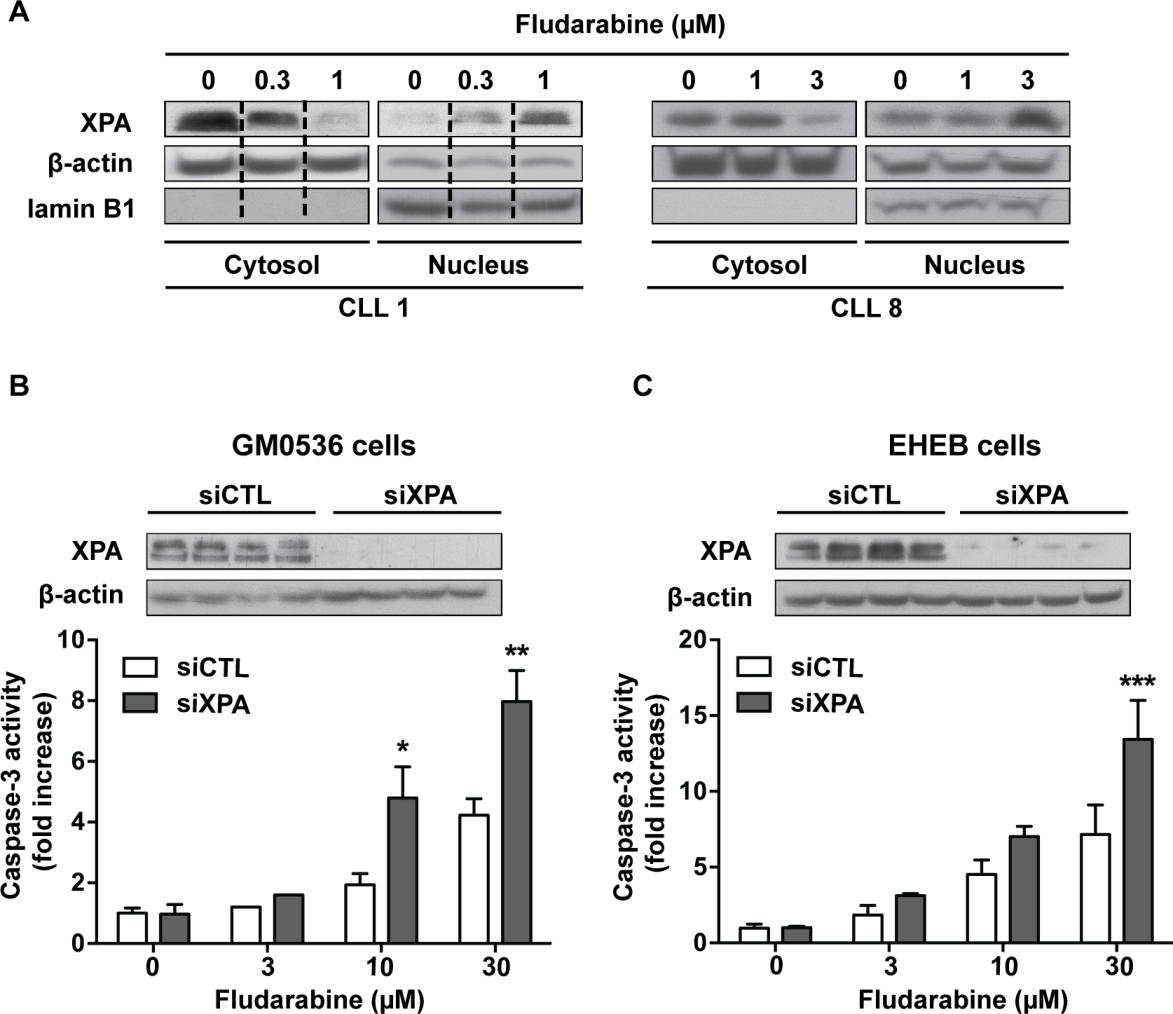
Involvement of XPA in cell sensitivity to fludarabine (**A**) CLL cells were incubated for 24 h with fludarabine at the indicated concentrations. Cytoplasmic and nuclear fractions were extracted and analyzed for XPA by Western blot. β-Actin and lamin B1, a specific nuclear protein, served as loading controls. Data shown for CLL1 were cropped from the same western blot image and combined as indicated by the dotted lines. **(B**) GM0536 and (**C**) EHEB cells were transfected with scrambled (siCTL) or XPA siRNA (siXPA) for 24 or 48 h, respectively. Then, cells were incubated for additional 24 h with fludarabine at the indicated concentrations. Upper panels show XPA levels analyzed by Western blot in siCTL-treated and siXPA-treated cells and lower panels show caspase-3 activity measured by a fluorometric assay. Results are means ± SEM of 3 independent experiments. Significance relative to siCTL-treated cells was analyzed by two-way Anova followed by Bonferroni post-test: *P < 0.05; **P < 0.01; ***P < 0.001.

To determine whether purine analogs could activate NER, we analyzed the effect of fludarabine on the DNA repair protein XPA (xeroderma pigmentosum, complementation group A). XPA is an important repair factor in NER, which was shown to be imported from the cytoplasm into the nucleus after DNA damage induced by UV [28] or alkylating drugs [29, 30]. As illustrated in Figure 5A, we found that fludarabine induced dose-dependent translocation of XPA from cytoplasm to nucleus, suggesting that NER can be activated in response to purine analogs.

### Down-regulation of XPA increases purine analog-induced apoptosis in cell lines

To determine if DNA damage repair by NER could limit the sensitivity of CLL cells to purine analogs, we used siRNA to down-regulate XPA. However, the cytotoxicity of electroporation procedure limited usefulness of primary CLL cells for these investigations. Therefore, we analyzed the effect of XPA silencing in lymphoblastoid cells (GM0536) and in the CLL cell line EHEB. Cells were transfected with scramble or XPA siRNA for 24 (GM0536) or 48 h (EHEB) and then incubated with fludarabine at increasing concentrations for additional 24 h. Relatively high concentrations of fludarabine were selected for these experiments due to the lower sensitivity of these cell lines to fludarabine with IC_50_ of 6.5 and 17.2 μM for GM0536 and EHEB cells, respectively. XPA protein was strongly down-regulated by XPA siRNA (Figure 5B, 5C, upper panels), which was concomitant with significantly higher caspase-3 activation in response to fludarabine (Figure 5B, 5C, lower panels).

## DISCUSSION

In this study, we have shown that aphidicolin markedly sensitizes CLL cells to fludarabine and cladribine, decreasing their IC_50_ by 4.5- and 2.8-fold, respectively. This finding contrasts with previous observations that aphidicolin reduced fludarabine cytotoxicity in leukemic cell lines, in which incorporation into DNA during replication is the key mechanism of cytotoxicity [31, 32]. In quiescent CLL cells, incorporation of purine analogs into DNA occurs through background DNA repair activity, most likely by the aphidicolin-insensitive DNA polymerase β [33], explaining why no inhibition of their incorporation into DNA, and therefore of their cytotoxicity, was recorded in the presence of aphidicolin.

At the molecular level, aphidicolin enhanced p53 phosphorylation and accumulation of γH2AX, an early marker of DNA damage [19], indicating that aphidicolin increases DNA damage in response to purine analogs. Since aphidicolin did not trigger H2AX phosphorylation *per se,* was without effect on the activation of purine analogs, and maximally inhibited DNA repair synthesis in quiescent lymphocytes at the concentration used in our experiments [34], we postulated that aphidicolin enhances purine analog-induced DNA damage by reducing DNA repair. In line with this hypothesis, disappearance of γH2AX, which was found to occur after removal of purine analogs and can be considered as readout of DNA repair, was significantly slowed down by aphidicolin.

The possibility to increase purine analog efficacy by preventing DNA repair has still been little explored. Nevertheless, sensitization of CLL cells to fludarabine has been reported in the presence of NU7441, which impairs the NHEJ pathway through inhibition of DNA-dependent protein kinase [35], and methoxyamine, a BER inhibitor [36]. In addition, cladribine cytotoxicity was increased in NER-deficient lymphoblasts compared to wild-type cells [37]. Our observation that aphidicolin impedes DNA repair after treatment by purine analogs demonstrates that the DNA damage induced by these drugs is repaired, at least partially, by a pathway that involves DNA polymerases δ/ε. Among the DNA repair pathways involving polymerases δ/ε, namely long-patch BER, MMR and NER, only the latter is thought to operate in non-dividing cells [23]. That NER could be the pathway inhibited by aphidicolin in CLL cells in response to purine analogs is supported by several observations: (1) the repair of UV-C-induced DNA damage, known to occur by NER, was inhibited by aphidicolin, (2) XPA, an essential factor for NER, was translocated from the cytosol to the nucleus after fludarabine, as previously observed after UV and alkylating agent treatment [28, 29, 38], and (3) silencing of XPA in cell lines, including a CLL cell line, resulted in higher caspase-3 activation in response to fludarabine. Moreover, using genome-wide expression profiling, we previously observed that four NER-associated genes (*PCNA*, *XPC, GADD45*, and *DDB2)* were among the most up-regulated genes in CLL cells after treatment with fludarabine or cladribine *in vitro* [39]. Upregulation of *XPC* was also recorded in response to fludarabine *in vivo* [40]. All these data strongly indicate that the NER pathway can be activated by purine analogs in CLL cells, resulting in decrease of their efficacy.

Important finding of this study is the ability of aphidicolin to sensitize CLL cells to purine analogs regardless of prognostic factors of patients, including relapse, *IGHV* status and 17p deletion. Drug resistance represents a major therapeutic challenge in management of CLL [41]. Regarding purine analogs, several factors may be involved such as reduced intracellular accumulation of their active form, due to impaired transport or metabolism, or aberrant expression of certain Bcl-2 family proteins [6]. However 17p deletion, 11q deletion, and/or *TP53* gene mutations, which impair the ATM/p53 signaling pathway and apoptosis in response to DNA damage, are among the best-documented factors of clinical resistance to purine analogs as well as to other genotoxic therapies [41, 42]. Strikingly, aphidicolin was able to synergize with purine analogs in all the 17p deleted cases investigated. Although the sample size was limited and p53 dysfunction might not be complete, these data suggest that increase of cell death induced by aphidicolin could occur by p53-dependent and -independent mechanisms. That DNA damage can induce signaling for apoptosis in a p53-independent manner has recently became increasingly evident from literature data in different types of cancer cells including the leukemic cell line HL-60 and primary CLL cells [35, 36, 43].

As inhibitor of DNA replication, aphidicolin possess strong antitumor activity in cancer cell lines *in vitro*. This property led to Phase I clinical trials with a water-soluble glycinate ester, which showed limited toxicity at concentrations achievable in humans, but also little antitumor effect [44]. Preclinical studies suggested that aphidicolin might be more effective in combination with platinum agents through inhibition of DNA repair elicited by these drugs [13]. However, clinical studies were not finalized, a major obstacle for clinical use being its low solubility and fast clearance from human plasma. Therefore, aphidicolin, as such, could not currently enter clinical trials in combination with fludarabine or cladribine, but studies are in progress to enable the development of novel aphidicolin derivatives with enhanced stability and inhibitory properties [45]. Regarding NER, many efforts have been made in last years to identify specific inhibitors of this pathway, but most so-called NER inhibitors appear to be nonspecific, have weak efficacy and are often toxic [46]. Last attempts pointed to find molecules that block interaction between essential components of the NER pathway, such as the interaction between ERCC1 and XPF, which is obligatory for the endonuclease activity of the complex. Novel inhibitors of this interaction have been recently identified and shown to enhance cytotoxicity of alkylating agents in cancer cell lines [47, 48].

In conclusion, our study indicates that purine analogs elicit a DNA repair pathway that involves the DNA polymerases δ/ε, most likely NER, and provides proof-of-concept that inhibition of this repair pathway could improve their therapeutic efficacy in CLL patients with a good, but also a worse prognosis. These finding should still increase interest in developing aphidicolin-like inhibitors and/or specific inhibitors of the NER pathway, which could be combined with fludarabine that remains a mainstay for CLL treatment.

## MATERIALS AND METHODS

### Drugs, reagents and antibodies

[8–^3^H]-Cladribine (7 Ci/mmol) and [8–^3^H]-fludarabine (4.4 Ci/mmol) were from Moravek Biochemicals (Brea, CA, USA). Fludarabine, 3-[4,5- dimethylthiazol-2-yl]-2,5-diphenyl-tetrazolium bromide (MTT) and propidium iodide were purchased from Sigma–Aldrich (St Louis, MO, USA). Cladribine was synthesized and supplied by Prof. J. Marchand (Laboratory of Organic Chemistry, Université catholique de Louvain, Belgium). Aphidicolin (APC) was from VWR (International Radnor, PA, USA). Ac-DEVD-AMC (Ac-Asp-Glu-Val-Asp-AMC) and AMC (7-amino-4-methyl-coumarin) were purchased from Alexis Biochemicals (San Diego, CA, USA). The annexin V-FITC apoptosis detection kit was from BD Biosciences (Franklin Lakes, NJ, USA). NU7026 was from Selleck (Houston, TX, USA). Antibodies used in this study were: anti-p53 (#sc126) from Santa Cruz Biotechnology (Santa Cruz, CA, USA); anti-p53-pS15 (#9284L), anti-caspase-3 (#9665S) and anti-caspase-9 (#9502S) from Cell Signaling Technologies (Beverly, MA, USA); anti-XPA (ab65963) and anti-lamin B1 (ab16048) from Abcam (Cambridge, UK); Alexa Fluor 488 Mouse anti-H2AX (pS139) from BD Biosciences; anti-β-actin from Sigma-Aldrich. Secondary antibodies (horseradish peroxidase conjugated anti-rabbit and mouse antibodies) were purchased from Sigma–Aldrich. Other chemicals, materials and reagents were from Sigma–Aldrich, Merck Biosciences (Gibbstown, NJ, USA) or Bio-Rad (Hercules, CA, USA) Laboratories.

### Patients and CLL cell isolation

Peripheral blood mononuclear cells were obtained from 34 different CLL patients, who had provided informed consent following protocol approval by the Ethics Committee of the Cliniques universitaires Saint-Luc (Brussels, Belgium). This study was conducted in accordance with the Declaration of Helsinki. All patients had an established diagnosis by standard morphological and immunological criteria [49], were free of any anticancer treatment for at least 3 months and had lymphocytes ≥ 30 × 10^9^/L. Patient characteristics are summarized in Supplementary Table 2. Conventional karyotype and interphase fluorescence *in situ* hybridization (FISH) analysis were performed as previously described [50]. Mononuclear cells (> 95% CLL cells, as confirmed by flow cytometry analysis) were isolated and cultured as described before [39]. When successive analyses were performed in samples from the same patient, they were separated by a period of at least one month. In combination studies, CLL cells were incubated with 3 μM aphidicolin for 30 min prior to the addition of fludarabine, cladribine or other chemotherapeutic drugs. Stock solutions of hydrophobic compounds were prepared in DMSO and equal amounts of DMSO were added in untreated and treated cells. The final DMSO concentration was ≤ 0.2%. In some experiments, cells were UV-C irradiated as described before [51] or subjected to IR using a ^137^Cs source at a dose rate of 2.43 Gy/min at room temperature.

### Cell viability and drug interaction analysis

To assess the influence of aphidicolin on the sensitivity of CLL cells to purine analogs, freshly isolated cells (2 × 10^6^ cells/well) were incubated in 96-well plates with increasing concentrations of fludarabine or cladribine in the absence or presence of 3 μM aphidicolin. After 96 h, CLL cell survival was measured using the MTT assay as previously described [52]. Each condition was done in triplicate in the same experiment. The concentrations of fludarabine or cladribine required to kill 50% of cells (IC_50_) were determined graphically. Interaction between aphidicolin and fludarabine or cladribine, i.e. synergism, additivity or antagonism, was defined according to the multiplicative model as previously reported [18].

### Western blot analysis

Cell protein extracts were obtained as reported [8]. Protein concentration was determined using the Bio-Rad protein assay. Equal amounts of denatured protein (usually 50 μg) were subjected to SDS-PAGE in 12% (w/v) polyacrylamide gels and transferred to Hybond^TM^-C Extra membranes (Amersham Biosciences). After blocking with 5% skim milk for 1 h at room temperature, the membranes were probed overnight at 4°C with the primary antibody (diluted 1/1000, or 1/10 000 for the anti-β-actin antibody). After washing, the membranes were incubated for 1 h at room temperature with horseradish peroxidase-conjugated secondary antibody (1/10 000). Proteins were visualized using Clarity Western ECL Substrate (Bio-Rad) and ECL chemiluminescence film (Fujifilm). Adobe Photoshop CS6 was used for image processing.

### Fludarabine and cladribine metabolism in intact CLL cells

The influence of aphidicolin on the metabolism of fludarabine or cladribine in CLL cells was analyzed as previously described, using ^3^H-labeled purine analogs [10].

### Isolation of nuclear and cytoplasmic fractions

Cell pellets (50 × 10^6^ cells) were resuspended in 80 μl of buffer A (10 mM HEPES, pH 7.9, 0.1 mM EDTA, 10 mM KCl, 1 mM dithiothreitol, 0.1% (v/v) Nonidet-P40 and freshly added protease inhibitors) and incubated for 20 min on ice with gentle shaking. After centrifugation at 7200 × g for 5 min, the supernatant containing cytoplasmic fraction was transferred to a new tube. The remaining insoluble fraction was washed once in Buffer A without NP-40 and then solubilized in 25 μl of buffer B (20 mM HEPES, pH 7.9, 1 mM EDTA, 50 mM KCl, 10 mM dithiothreitol, 1% (v/v) Triton X-100 and protease inhibitors) and incubated 30 min on ice. The supernatant containing nuclear fraction was obtained after centrifugation at 16 000 × g for 10 min.

### γH2AX flow cytometry analysis

CLL samples (1 × 10^6^ cells) were fixed in 70% ethanol and maintained at −20°C for at least 1 h. Next, cells were washed twice with cold PBS, and rehydrated for 10 min at 4°C in buffer containing PBS, 4% FBS and 0.1% Triton. After centrifugation, cells were resuspended in 100 μl of the same buffer with the Alexa Fluor 488 Mouse anti-H2AX (pS139) antibody (1/200), incubated for 1 h at room temperature in darkness, washed and resuspended in PBS. Labeled cells were analyzed with FACSVerse flow cytometer (BD Biosciences).

### Apoptosis assays

Apoptosis was quantified by Annexin V-FITC/propidium iodide (PI) double staining (Beckton Dickinson) followed by analysis using a FACSVerse flow cytometer. Annexin-V positive and PI negative cells were defined as early apoptotic, while Annexin-V and PI dual positive cells were considered as apoptotic/necrotic. As incubation of CLL cells in fresh medium resulted in spontaneous apoptosis (5 to 20% dead cells after 48 h), we calculated specific drug-induced apoptosis as described elsewhere [53] using the following formula: 100 × [experimental apoptosis (%) − spontaneous apoptosis (%)/[100% − spontaneous apoptosis (%)]. Caspase-3 activity was determined by a fluorometric assay with Ac-DEVD-AMC as a fluorogenic substrate using samples containing 50 × 10^6^ CLL cells, as previously reported [10]. Procaspase-9 and procaspase-3 were analyzed by Western blot.

### RNA interference in cell lines

The cell lines used for RNA interference experiments were the EBV-immortalized lymphoblastoid cell line GM0536, obtained from the NIGMS Human Mutant Cell Repository (Camden, NJ, USA), and the CLL cell line EHEB, purchased from DSMZ-German Collection of Microorganisms and Cell Culture (Braunschweig, Germany). Cells were cultured as described previously [54] and transfected using Amaxa^™^ nucleofector and nucleofection Kit V (Amaxa, Cologne, Germany) according to the Amaxa guidelines. Briefly, 2 × 10^6^ cells were resuspended in 100 μl of the Nucleofector solution V. Targeting or not-targeting small interfering RNAs (siRNAs) were added at a concentration of 900 nM to the cell suspensions and the mixtures were transferred to the Amaxa certified cuvettes. Nucleofection was performed using the M-013 program. Experiments were conducted 24 or 48 h after transfection, for GM0536 or EHEB cells, respectively. siRNA duplexes were from Dharmacon (Lafayette, CO, USA): XPA siRNA (SMARTpool: ON-TARGETplus XPA siRNA) and the non-targeting control (ON-TARGETplus Non-targeting Pool).

### Statistical analysis

Results obtained in at least 3 independent experiments were normalized and expressed as the means ± SEM. Significance was analyzed by different statistical methods as detailed in the figure legend. Changes were considered significantly different when *P* < 0.05.

## SUPPLEMENTARY MATERIALS FIGURES AND TABLES


