# Supplementary material for: Targeting DNA repair with aphidicolin sensitizes primary chronic lymphocytic leukemia cells to purine analogs
Source: Oncotarget. 2016 May 20;7(25):38367–79. doi: 10.18632/oncotarget.9525 (PMC5122396; doi:10.18632/oncotarget.9525)
Supplement: Supplementary file 1 [file oncotarget-07-38367-s001.pdf]

Targeting DNA repair with aphidicolin sensitizes primary chronic lymphocytic leukemia cells to purine analogs

Supplementary Materials

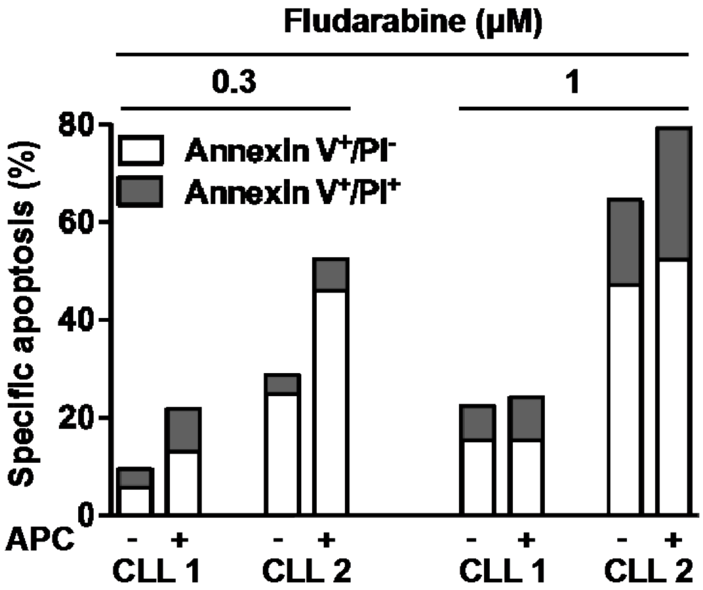

Supplementary Figure S1: Aphidicolin enhances fludarabine-induced apoptosis. Apoptosis was measured by Annexin V-binding assay in CLL cells from two patients after a 48 h-incubation in the absence or presence of 3 µM aphidicolin (APC) with or without fludarabine at the indicated concentrations. Cells undergoing apoptosis are Annexin V^+/PI−, while cells already dead are Annexin V^+/PI^+. Specific drug-induced apoptosis was expressed as explained in the Methods section of the paper.

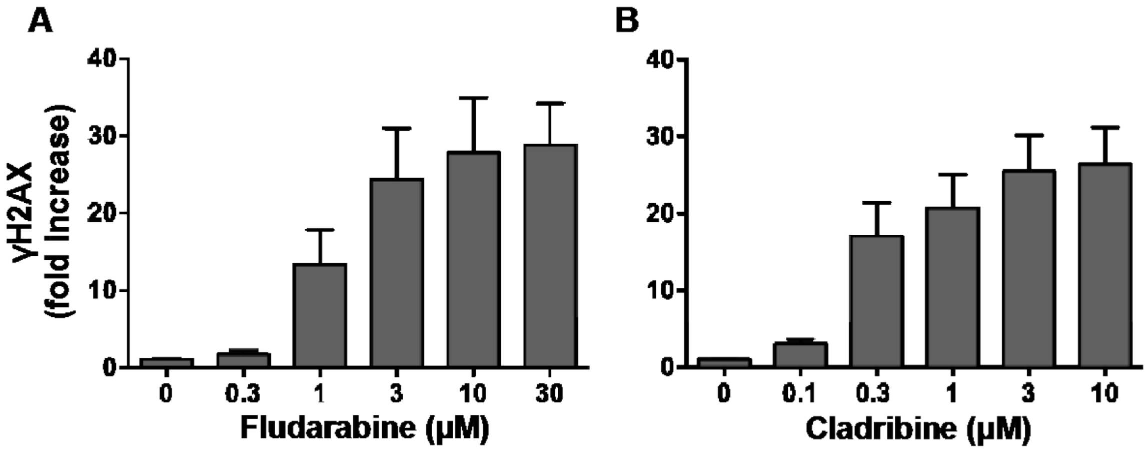

Supplementary Figure S2: γH2AX accumulation induced by purine analogs. γH2AX was measured by flow cytometry in CLL cells incubated for 24 h in the presence of fludarabine (**A**) or cladribine (**B**) at increasing concentrations. Results are means ± SEM of 5 independent experiments.

Supplementary Table S1: Influence of aphidicolin on the cytotoxicity of non-nucleosidic drugs

Drugs (*n*)	IC_{50} (μM)		Sensitization ratio (−APC/+APC)
	−APC	+APC	
Mafosfamide (5)	17.3 ± 7.3	3.0 ±1.3	5.8***
Doxorubicin (3)	8.0 ± 2.6	2.1 ± 0.3	3.8*
Nutlin-3a (3)	4.9 ± 1.2	3.5 ± 0.9	1.4^{ns}

CLL cells were incubated with increasing concentrations of the indicated drugs in the presence or absence of 3 μM aphidicolin (APC). After 96 h, cell survival was analyzed using the MTT assay. IC_{50} values were determined as in Figure 1. Results are means ± SEM of 5 or 3 experiments as indicated. Significance relative to the absence of APC was analyzed by paired *t*-test: *$P < 0.05$; ***$P < 0.001$; ns, not significant.

Supplementary Table S2: Biological and clinical characteristics of patients (*n* = 34)

Characteristics	Data
Age (years)	
Median	71
Range	48–88
Gender	
Female	15
Male	19
Stage	
Binet A	19
Binet B	10
Binet C	5
IGHV	
Mutated	13
Unmutated	5
Not determined	16
FISH*	
Normal	2
17p deletion	6
11q deletion	1
Trisomy 12	2
13q deletion	16
Not determined	7
CD38	
Positive (≥ 30%)**	1
Negative (< 30%)	26
Not determined	7
Previous treatment	
Yes	13
No	21
Karyotype	
Normal	14
Abnormal	18
Not determined	2

*According to Döhner's categories [1].
**Positive CD38 status refers to CD38 expression on 30% or more of CLL cells.

REFERENCES

1. Dohner H, Stilgenbauer S, Benner A, Leupolt E, Krober A, Bullinger L, Dohner K, Bentz M, Lichter P. Genomic aberrations and survival in chronic lymphocytic leukemia. N Engl J Med. 2000; 343:1910–1916.
